# Role of Extracranial Carotid Duplex and Computed Tomography Perfusion Scanning in Evaluating Perfusion Status of Pericarotid Stenting

**DOI:** 10.1155/2016/7051856

**Published:** 2016-03-08

**Authors:** Chih-Ming Lin, Yu-Jun Chang, Chi-Kuang Liu, Cheng-Sheng Yu, Henry Horng-Shing Lu

**Affiliations:** ^1^Stroke Center, Department of Neurology, Changhua Christian Hospital, Changhua and Graduate Institute of Biological Science and Technology, National Chiao Tung University, Hsinchu, Taiwan; ^2^Epidemiology and Biostatistics Center, Changhua Christian Hospital, Changhua, Taiwan; ^3^Department of Medical Imaging, Changhua Christian Hospital, Changhua, Taiwan; ^4^Institute of Statistics, National Chiao Tung University, 1001 Ta Hsueh Road, Hsinchu 30010, Taiwan

## Abstract

Carotid stenting is an effective treatment of choice in terms of treating ischemic stroke patients with concomitant carotid stenosis. Though computed tomography perfusion scan has been recognized as a standard tool to monitor/follow up this group of patients, not everyone could endure due to underlying medical illness. In contrast, carotid duplex is a noninvasive assessment tool and could track patient clinical condition in real time. In this study we found that “resistance index” of the carotid ultrasound could detect flow changes before and after the stenting procedure, thus having great capacity to replace the role of computed tomography perfusion exam.

## 1. Introduction

Stroke is the fourth leading cause of death worldwide [1]. Among its various etiologies, carotid stenosis is the most well observed and accounts for 30 to 35 percent of total ischemic strokes [2]. Stenosis grading exceeding 70% lumen reduction doubles or triples the risk of stroke and its recurrence. Vascular neurologists have reported that prompt management of carotid stenosis through standard carotid stenting prevents stroke and its recurrence. In the prestenting phase, doctors routinely administer computed tomography (CT) perfusion scans to study the cerebral blood perfusion condition in the bilateral cerebral hemispheres.

Although CT perfusion scanning has several advantages and has been recognized as a standard tool for assessing cerebral perfusion in patients undergoing stenting, not all patients can tolerate it. This neuroimaging tool entails the use of a contrast medium, which is hazardous to patients, especially those with impaired renal function.

Extracranial carotid ultrasounding is noninvasive and requires no contrast mediums. Investigation and validation of the role of ultrasound in evaluating flow conditions in pre- and poststenting phases are yet to be reported in the literature. This study utilizes a clinical parameter—resistance index (RI)—generated through carotid ultrasound exams and CT perfusion scans to investigate whether flow changes can be simultaneously observed in these two examinations.

## 2. Materials and Methods

### 2.1. Patient Identification

We consecutively recruited 15 patients scheduled to undergo carotid stenting. The patients were admitted to our outpatient clinics and emergency departments or transferred from our branch hospital; all patients had been hospitalized for examination and treatment. We included patients with age ≥18 years, with an initial ischemic or recurrent strokes, with angiographic evidence of >70% carotid stenosis, with no other etiology of stroke that could explain the index event, and with no evidence of recurrent stroke during the study period and followed up at least 6 months after the stenting treatment. Exclusion criteria were patients with cerebral hemorrhage, cerebral arteriovenous malformations, aneurysms, and bilateral moderate-severe carotid stenosis, and less than 6 months' follow-ups. The enrolled patients were hospitalized for medical treatments along with baseline biochemistry work-ups. The ischemic stroke was confirmed by the diffusion weighted sequence of magnetic resonance imaging. The diagnostic digital subtraction angiography (DSA) was arranged during the hospitalization to gauge the degree of the carotid stenosis. The patients were stented one month after the index episode (stroke event). The participants were administered extracranial carotid ultrasound and CT angiography/perfusion (CTA/P) scanning simultaneously before carotid stenting. The above mentioned examinations were repeated simultaneously one month after stenting. This study was approved by the Institutional Review Board of Changhua Christian Hospital.

### 2.2. Baseline Clinical Characteristics

Baseline demographic data and clinical characteristics were collected including age, gender, and body mass index (BMI); baseline biochemistry data were collected on admission, such as low density lipoprotein (LDL) and glycated hemoglobin (HbA1c) levels and evidence or any major past history. Performance in activities of daily living was measured before stenting with the Barthel Index.

### 2.3. Cervical Carotid Ultrasound Examination

Cervical carotid artery examination was performed in our ultrasonography laboratory by using a Philips iE33 7-Mhz linear transducer. Patients slightly tilted their head contralaterally, and the transducer was placed on their necks. First, cross-sectional B-mode scanning and longitudinal screening were performed to identify and confirm intraluminal plaques, respectively. Peak systolic velocity (PSV), end diastolic velocity (EDV), and resistance index (RI) of the CCA, internal carotid artery (ICA), and external carotid artery (ECA) were measured. RI is given by PSV − EDV/PSV. The degree of carotid stenosis was calculated using the European Carotid Surgery Trial method [3].

### 2.4. Computed Tomography Angiography/Perfusion Scan (CTA/P Imaging)

CTA examinations were performed using a second-generation dual-source CT scanner (SOMATOM Definition Flash, Siemens Healthcare, Forchheim, Germany). Perfusion data sets were postprocessed using a Siemens Multimodality Workplace Workstation (Siemens Medical, Germany), which calculated mean transit time (MTT), cerebral blood volume (CBV), cerebral blood flow (CBF), and time to peak (TTP). The arterial input and venous outflow curves were analyzed to ensure data set completeness. The CTP parameters are defined as follows:

1. dMTT: ipsilateral MTT − contralateral MTT.
2. MTT ratio: ipsilateral MTT/contralateral MTT.
3. MTT index: (ipsilateral MTT − contralateral MTT)/contralateral MTT.
4. dCBV: ipsilateral CBV − contralateral CBV.
5. CBV ratio: ipsilateral CBV/contralateral CBV.
6. CBV index: (ipsilateral CBV − contralateral CBV)/contralateral CBV.
7. dCBF: ipsilateral CBF − contralateral CBF.
8. CBF ratio: ipsilateral CBF/contralateral CBF.
9. CBF index: (ipsilateral CBF − contralateral CBF)/contralateral CBF.
10. dTTP: ipsilateral TTP − contralateral TTP.
11. TTP ratio: ipsilateral TTP/contralateral TTP.
12. TTP index: (ipsilateral TTP − contralateral TTP)/contralateral TTP.

### 2.5. Magnetic Resonance Imaging and Angiography (MRI/A)

Structural and functional MR imaging and angiographic examinations were performed using a 3-T (Magnetom Verio, Siemens Healthcare, USA) or a 1.5-T imager (Magnetom Aera, Siemens Healthcare) with a cervical coil. Standard protocol to evaluate a stroke including axial DWI, apparent diffusion coefficient, and fluid-attenuated inversion-recovery sequences was followed. Contrast-enhanced MR angiography was not routinely performed.

### 2.6. Digital Subtraction Angiography (DSA) and Stenting

**Figure 1:**
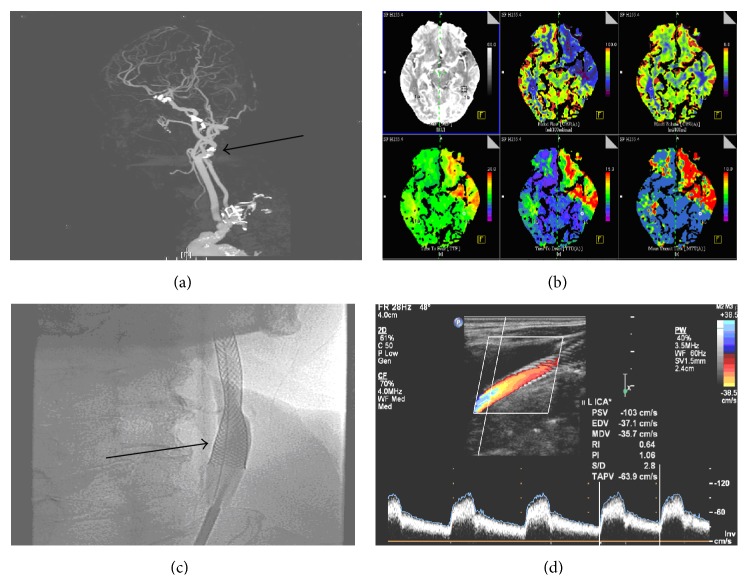
An example of left side severe internal carotid artery stenosis undergoing carotid stenting treatment. An 85-year-old male patient presented with right side hemiparesis and aphasia 24 hours before admission to our neurology ward. (i) (a) denotes CTA scan showing >70% lumen reduction of left side proximal internal carotid artery and distal part of common carotid artery (black arrow). (ii) (b) shows the CT perfusion scan that suggests decreased blood perfusion to the left cerebral hemisphere indicating critical blood flow demand compared to the right side cerebral hemisphere. (iii) (c) presents the poststenting status with umbrella device that prevent embolus distal migration causing major subsequent stroke event (black arrow). (iv) (d) displays the follow-up cervical carotid ultrasound of normalized flow profile.

Biplanar intra-arterial DSA was performed using a biplanar flap panel rotational angiography unit (Axiom Artis Zee, Siemens Healthcare) with an image intensifier matrix of 1024 × 1024 pixels and a final pixel size of 0.37 mm. A self-expandable carotid wallstent (7 mm × 30 mm) was delivered coaxially through the guiding catheter into the stenotic area (Figure 1).

### 2.7. Statistical Analyses

Continuous variables are presented as mean ± standard deviation (SD), median, percentile, minimal, and maximal values. Categorical variables are presented as numbers and percentages. Pre- and poststenting CT perfusion and carotid ultrasound variables were compared using Wilcoxon signed ranks test. *P* < 0.05 was considered statistically significant. All statistical analyses were performed using SPSS for Windows (Version 16.0, SPSS Inc., Chicago, IL, USA).

## 3. Results

The baseline clinical characteristics of the 15 patients are summarized in Tables 1 and 2. Tables 3 and 4 report the flow changes reflected by the parameters of CT perfusion and carotid ultrasound examinations.

Ipsilateral mean values of MTT, CBV, and TTP decreased after stenting, whereas CBF increased slightly. The majority of patients exhibited decreased MTT, CBV, and TTP. Pre- and posttreatment TTP values differed significantly (*P* = 0.031, <0.05). Contralateral MTT, CBV, and TTP decreased, whereas the mean CBF increased slightly. The ratio of patients exhibiting decreased and increased contralateral MTT, CBV, CBF, and TTP values is approximately 1 (Table 3).

Ipsilateral RI analyses showed that mean CCA RI reduced from 0.78 to 0.75, and most patients exhibited decreased CCA RIs. By contrast, mean ICA RI increased from 0.62 to 0.69, and most patients exhibited increased ICA RIs. Mean ECA RI decreased from 0.84 to 0.80; there are five and six patients, demonstrating decreased and increased ECA RIs, respectively (Table 3).

Contralateral RI analyses revealed that CCA, ICA, and ECA RIs increased after stenting, with most patients showing increased mean CCA and ICA values; CCA RI increased from 0.73 to 0.77, ICA RI increased from 0.67 to 0.70, and ECA increased from 0.87 to 0.88 (Table 3).

The mean values of all other 12 CT perfusion scan parameters (Table 4) decreased after stenting. The majority of patients showed decreased values. Among the 12 parameters, MTT ratio, MTT index, and TTP ratio decreased significantly (*P* = 0.013, 0.039, and 0.017, <0.05).

## 4. Discussion

Stroke is the fourth leading cause of death worldwide [1, 2], and, among its various etiologies, carotid stenosis accounts for the majority of strokes. Carotid stenting can effectively prevent stroke and its recurrence.

CT perfusion is a standard assessment routinely performed before carotid stenting because it provides vivid color images and several parameters that clarify the ipsilateral and contralateral cerebral hemispheres perfusion status, as well as identifying stroke location [4–9]. However, not all patients can undergo this assessment tool. This is because they entail the use of contrast mediums, which is hazardous to patients with impaired renal function. In addition, CT perfusion administration is restricted to medical facilities, thus requiring patients to visit the facility. Moreover, it requires multiple operators, making it time intensive and expensive for patients.

By contrast, carotid duplex is a mobile, single-operator, contrast-medium-free, and inexpensive examination. Although no studies have examined the role of RI in evaluating cerebral perfusion status after carotid stenting, RI has been widely used in nephrology. Derchi et al. tested RI in patients with renal dysfunction and reported that the risk of renal impairment increased twofold when renal RI was >0.63 [10]. In addition, RI is effective in predicting kidney transplant outcomes [11–13].

CT perfusion, by convention, is used to assess the cerebral artery perfusion status. The improvement of cerebral blood perfusion after stenting treatment can be indicated by a reduced MTT and an incased CBF within the ipsilateral carotid system [14, 15]. Our study is in accordance with these statements. The findings of MTT and CBF over the contralateral carotid system in the published papers [2, 16], however, have not come to general consensus. Our study shows that the MTT and CBF values decreased after treatment. Moreover, the absolute difference, ratio, and index values of bilateral cerebral hemispheres are also calculated. The results show a drop in values, particularly within the MTT ratio, MTT index, and TTP ratio.

RI, a clinical parameter generated from carotid ultrasounding, represents the general downstream blood vascular bed resistance level [17]. RI > 0.75 denotes increased downstream vascular bed, which can be due to various factors, including obstructions. Because CCA and ICA supply the majority of blood to the intracranial hemispheres, their RI values are lower than that of ECA in normal circumstances (CCA and ICA < 0.75, ECA > 0.75).

**Figure 2:**
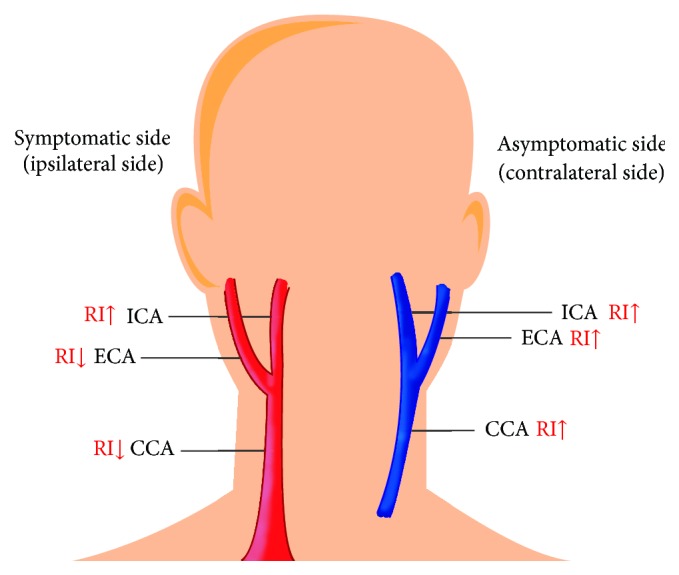
RI and flow changes of the patients receiving carotid stenting treatment. RI changes within ipsilateral and contralateral carotid systems. Red color denotes increased blood flow and blue color decreased flow.

In our study, ipsilateral mean CCA RI values decreased from 0.78 to 0.75 after carotid treatment, indicating that ICA flow smoothened and resistance level decreased after treatment, subsequently drawing more blood to perfuse the same side of the cerebral hemisphere. The mean ipsilateral ICA RI value, however, rose from 0.62 to 0.69, elucidating the vasoconstriction mechanism after upstream dilation. Nevertheless, ipsilateral CCA and ICA returned to their normal values (<0.75). Conversely, on the contralateral side of the carotid system, mean RI rose in all carotid arteries (CCA, CA, and ECA), which explains the relatively low blood flow to the contralateral side of the vasculature because most of the blood was supplied to the ipsilateral side after stenting (Figure 2).

Our study has a few limitations. First, with only 15 patients, the sample size is small; the results must be interpreted cautiously, and additional studies are necessary for confirming their applicability in various other conditions. Second, the carotid duplex and CT perfusion scans were conducted during peristenting phases. Although the immediate effect of stenting can be detected, the long-term cerebral perfusion status is yet to be investigated. Finally, all participants in this study were of Asian origin; therefore, the results can be generalized only to an Asian population.

## 5. Conclusions

The findings of our RI study on carotid ultrasounding and CT perfusion scanning can provide important clinical information in evaluating the perfusion status in patients receiving stenting, especially if the patient condition is not suitable for repeated CT perfusion examinations.

## Supplementary Material

The standard protocol of computed tomography angiography/perfusion, magnetic resonance imaging and angiography, along with digital subtraction angiography for carotid stenting procedure of Changhua Christian Hospital.

## Figures and Tables

**Table 1:** Baseline patient features.

(*n* = 15)	Mean	SD	Median	Percentile 25	Percentile 75	Min	Max
Age	66.47	9.59	69.00	56.00	73.00	47.00	79.00
Grade of stenosis	0.71	0.10	0.74	0.67	0.78	0.53	0.84
SBP	137.27	10.73	138.00	128.00	149.00	118.00	155.00
DBP	77.47	8.33	80.00	74.00	84.00	62.00	90.00
Weight	64.36	10.22	63.50	55.00	68.50	48.00	86.00
Height	163.47	8.33	165.00	162.00	170.00	144.00	172.00
BMI	24.09	3.36	23.56	20.78	28.20	18.34	29.07
Barthel	87.50	10.14	87.50	80.00	100.00	75.00	100.00
LDL	121.14	29.41	121.00	97.90	134.00	86.40	175.00
HbA1c	6.94	1.89	6.65	5.70	7.30	5.30	12.60
Ac sugar	119.67	46.18	96.00	94.00	128.00	80.00	227.00
Uric acid	6.25	1.43	6.35	5.40	7.00	4.10	9.20

*Note*. Min, minimum; Max, maximum; SBP, systolic blood pressure; DBP, diastolic blood pressure; BMI, body mass index; LDL, low density lipoprotein; SD, standard deviation.

HbA1c, glycated hemoglobin; AC sugar, fasting blood sugar.

**Table 2:** Baseline patient demographics.

	*N*	%
Gender		
Female	2	13.3
Male	13	86.7
Diabetes mellitus		
No	6	40.0
Yes	9	60.0
Hypertension		
No	3	20.0
Yes	12	80.0
Hyperlipidemia		
No	4	26.7
Yes	11	73.3
Prior stroke		
No	8	53.3
Yes	7	46.7

**Table 3:** Changes in carotid duplex and CT perfusion parameters after carotid stenting.

	(*n* = 15)	Ipsilateral												Contralateral											
		*N*	Mean	SD	Median	*Q*_1_	*Q*_3_	Min	Max	Change status (number)				*N*	Mean	SD	Median	*Q*_1_	*Q*_3_	Min	Max	Change status (number)			
	Time									Decrease	Increase	The same	*P* value^a^									Decrease	Increase	The same	*P* value^a^
MTT	Before treatment	13	7.06	3.33	6.27	4.29	10.16	3.28	13.06					13	5.74	2.93	5.05	3.59	7.77	3.05	12.07				
	After treatment	13	5.46	4.32	3.74	3.51	5.58	3.09	18.66	10	3	0	0.101	13	4.78	2.34	4.15	3.27	5.44	3.16	11.81	7	6	0	0.382

CBF	Before treatment	15	39.89	17.15	42.56	24.50	56.93	13.93	61.56					15	41.62	18.84	38.74	24.41	58.96	11.46	76.91				
	After treatment	15	40.65	14.01	39.74	34.90	48.08	5.17	62.60	8	7	0	1.000	15	43.56	20.40	40.78	35.57	47.89	7.86	106.83	7	8	0	0.776

CBV	Before treatment	15	3.02	0.61	2.93	2.66	3.38	1.98	4.39					15	3.00	1.17	3.01	1.96	3.32	1.73	5.79				
	After treatment	15	2.63	1.23	2.33	2.15	2.87	1.57	6.73	11	4	0	0.061	15	2.62	1.02	2.38	2.26	2.60	1.38	5.91	9	6	0	0.211

TTP	Before treatment	15	18.89	10.50	12.78	10.08	28.65	8.44	39.28					15	17.91	10.21	10.66	9.22	26.85	8.76	35.30				
	After treatment	15	12.17	4.13	11.12	9.32	12.70	9.18	23.60	11	4	0	0.031	15	12.50	2.80	11.71	10.82	13.57	9.72	21.03	9	6	0	0.307

CCA RI	Before treatment	12	0.78	0.14	0.80	0.71	0.89	0.43	0.92					13	0.73	0.06	0.75	0.69	0.77	0.60	0.82				
	After treatment	12	0.75	0.08	0.76	0.76	0.79	0.55	0.88	8	2	2	0.202	13	0.77	0.09	0.76	0.74	0.83	0.64	0.92	4	7	2	0.075

ICA RI	Before treatment	12	0.62	0.19	0.62	0.52	0.73	0.24	0.91					11	0.67	0.10	0.70	0.56	0.75	0.52	0.84				
	After treatment	12	0.69	0.10	0.70	0.60	0.76	0.58	0.89	3	7	2	0.103	11	0.70	0.07	0.69	0.63	0.78	0.57	0.81	3	7	1	0.220

ECA RI	Before treatment	13	0.84	0.12	0.85	0.80	0.91	0.53	1.00					13	0.87	0.11	0.90	0.80	0.96	0.61	1.00				
	After treatment	13	0.80	0.25	0.85	0.79	0.94	0.00	1.00	5	6	2	0.893	13	0.88	0.09	0.91	0.81	0.96	0.75	1.00	6	6	1	0.409

*Note*. SD, standard deviation; CCA, common carotid artery; ICA, internal carotid artery; ECA, external carotid artery; RI, resistance index; MTT, mean transit time; CBF, cerebral blood flow; CBV, cerebral blood volume; TTP, time to peak.

*Q*_1_: percentile 25; *Q*_3_: percentile 75.

^a^ *P* value by Wilcoxon signed ranks test.

**Table 4:** CT perfusion parameters before and after stenting.

	Time																			
	Before treatment								After treatment								Change status (number)			
	*N*	Mean	SD	Median	*Q*_1_	*Q*_3_	Min	Max	*N*	Mean	SD	Median	*Q*_1_	*Q*_3_	Min	Max	Decrease	Increase	The same	*P* value
MTT ratio	13	1.33	0.50	1.19	1.04	1.76	0.44	2.27	13	1.10	0.40	1.01	0.80	1.35	0.65	2.06	11	2	0	0.013
dMTT	13	2.43	2.37	1.64	0.56	3.53	0.03	7.30	13	1.59	1.92	0.74	0.35	2.30	0.03	6.85	9	3	1	0.182
MTT index	13	0.44	0.41	0.29	0.12	0.76	0.01	1.27	13	0.29	0.29	0.23	0.09	0.41	0.01	1.06	11	2	0	0.039

CBV ratio	15	1.11	0.36	1.04	0.92	1.28	0.50	1.99	15	1.01	0.20	0.98	0.86	1.14	0.64	1.42	9	6	0	0.394
dCBV	15	0.72	0.77	0.41	0.18	0.97	0.12	2.89	15	0.39	0.29	0.35	0.19	0.45	0.03	0.95	9	6	0	0.191
CBV index	15	0.26	0.26	0.14	0.06	0.49	0.04	0.99	15	0.15	0.12	0.14	0.08	0.20	0.01	0.42	9	6	0	0.334

CBF ratio	15	1.13	0.76	0.89	0.72	1.47	0.32	3.53	15	0.97	0.27	0.96	0.83	1.11	0.58	1.68	8	7	0	0.910
dCBF	15	13.91	15.00	8.54	5.69	18.19	1.26	52.41	15	9.13	11.97	5.10	2.69	6.94	1.32	44.94	10	5	0	0.100
CBF index	15	0.44	0.62	0.28	0.11	0.51	0.02	2.53	15	0.20	0.18	0.13	0.05	0.34	0.03	0.68	9	6	0	0.156

TTP ratio	15	1.08	0.18	1.01	0.97	1.32	0.79	1.37	15	0.96	0.14	0.92	0.86	1.04	0.78	1.26	12	3	0	0.017
dTTP	15	2.10	2.19	0.96	0.29	3.39	0.17	7.02	15	1.62	1.12	1.50	0.54	2.53	0.17	3.90	8	7	0	0.532
TTP Index	15	0.14	0.14	0.09	0.02	0.32	0.01	0.37	15	0.12	0.08	0.12	0.06	0.21	0.01	0.26	6	9	0	0.910

*Note*. SD, standard deviation; dMTT, difference of mean transit time; dCBV, difference of cerebral blood volume; dCBF, difference of cerebral blood flow; dTTP, difference of time to peak.

*Q*_1_: percentile 25; *Q*_3_: percentile 75.

*P* value by Wilcoxon signed ranks test.
