# Supplementary material for: Role of Extracranial Carotid Duplex and Computed Tomography Perfusion Scanning in Evaluating Perfusion Status of Pericarotid Stenting
Source: Biomed Res Int. 2016 Mar 8;2016:7051856. doi: 10.1155/2016/7051856 (PMC4802025; doi:10.1155/2016/7051856)
Supplement: Supplementary file 1 — The standard protocol of computed tomography angiography/perfusion, magnetic resonance imaging and angiography, along with digital subtraction angiography for carotid stenting procedure of Changhua Christian Hospital. [file 7051856.f1.pdf]

Supplementary Material

1. Computed tomography angiography/perfusion scan (CTA/P imaging)

After placing an 18-gauge intravenous catheter in the antecubital vein, 100 mL of Iodixanol (Vaque 320, GE Healthcare, Carrigtwohill, Ireland) or Iohexol (Omnipaque 350, GE Healthcare, Ireland) was infused as the contrast medium at a rate of 5 mL/s. The initial injection delay was estimated using the bolus-tracking technique, where the threshold was 100 Hounsfield units. Scanning was performed using a dual-energy mode with pitch, rotation time, and collimation of 0.9, 0.28 s, and 2 mm × 32 mm × 0.6 mm, respectively, at 100 kV/150 ref mAs (Tube A) and Sn140 kV/178 ref mAs (Tube B). The area from the aortic arch to the top of the neurocranium was scanned. CT images were reconstructed at a slice thickness of 0.6 mm and increment of 0.3 mm with a medium–smooth kernel. CTP scans were subsequently performed with a contrast bolus of 50 mL of Omnipaque 350 (GE Healthcare, Milwaukee, WI, USA)

2. Magnetic resonance imaging and angiography (MRI/A)

Three-dimensional TOF MR angiography without contrast enhancement was performed in the transverse plane using a sliding interleaved kY acquisition sequence comprising 6 overlapping slabs of 11 sections and the following parameters: section thickness, 1.2 mm; repetition time

(milliseconds)/echo time (milliseconds), 242/7; flip angle, 20°; field of view, 200 × 200 mm; matrix, 205 × 320. The final pixel size was 0. 975 mm × 0.625 mm. The entire imaging time was approximately 7 minutes.

3. Digital subtraction angiography (DSA) and stenting

Immediately after approaching the femoral artery, a 7-F catheter (Boston Scientific, Mach 1) was inserted into the right or left CCAs near the bifurcation. Posteroanterior and lateral projections were acquired at the level of the carotid bifurcation. A third oblique-angle projection was acquired if overlapping vessels were noted in the first 2 projections. For each projection, 11 mL of nonionic iodinated contrast medium (Omnipaque 350; GE Healthcare, Ireland) was intraarterially injected at a flow rate of 7 mL/s using an automatic injector (Mark V ProVis; Medrad). Subsequently, stenting was performed. A guidewire was inserted into the carotid artery on either side of the stenotic region. An EZ filter wire was used to prevent complication caused by distal embolic migration. Overall, 5000 units of prophylactic heparin were administered.
